# Diffuse pulmonary lymphangiomatosis: A rare case report in an adult

**DOI:** 10.1097/MD.0000000000017349

**Published:** 2019-10-25

**Authors:** Wencheng Yu, Liyun Mi, Jinpeng Cong, Wei Cheng, Yunqing Chen, Haihong Gong

**Affiliations:** a Department of Respiratory Medicine; b Department of Pathology, Affiliated Hospital of Qingdao University, Qingdao City, Shandong, China.

**Keywords:** adult, bronchoscopy, case report, diffuse pulmonary lymphangiomatosis, surgical intervention

## Abstract

**Rationale:**

Diffuse pulmonary lymphangiomatos (DPL) is a rare aggressive lymphatic disorder characterized by proliferation of anastomozing lymphatic vessels and extremely rare in adult patients.

**Patient concerns:**

We report a case of diffuse pulmonary lymphangiomatosis in 59-year-old man presented with cough and sputum for 2 months.

**Diagnoses:**

Combining clinical manifestations with results of radiological, bronchoscopy, and surgical lung biopsy, it was consistent with the diagnosis of DPL.

**Interventions:**

After bronchoalveolar lavage and biopsy, symptom of cough got worse suddenly accompanied by excessive chyloptysis. The patient received an emergency surgical intervention and low fat medium chain fat treatment.

**Outcomes:**

The patient was discharged with a much better health condition.

**Lessons:**

This case report is the oldest patient reported in the English literature, to the best of our knowledge. Serious complications of bronchoscopy should be considered, especially in DPL patients with severely enlarged mediastinum or with thin-walled translucent vesicles under endoscopy.

## 1 Introduction

Lymphangioma is an uncommon benign lymphatic tumor^[1]^ that occurs mostly in children with an equal sex prevalence.^[2]^ Diffuse pulmonary lymphangiomatos (DPL) is extremely rare in adult patients.^[3]^ The definite diagnosis mainly depends on the pathology. We report a confirmed case of DPL in 59-year-old man involving lung lesions, mediastinal and pleural effusions who was treated successfully with surgery.

## 2 Case report

**Figure 1:**
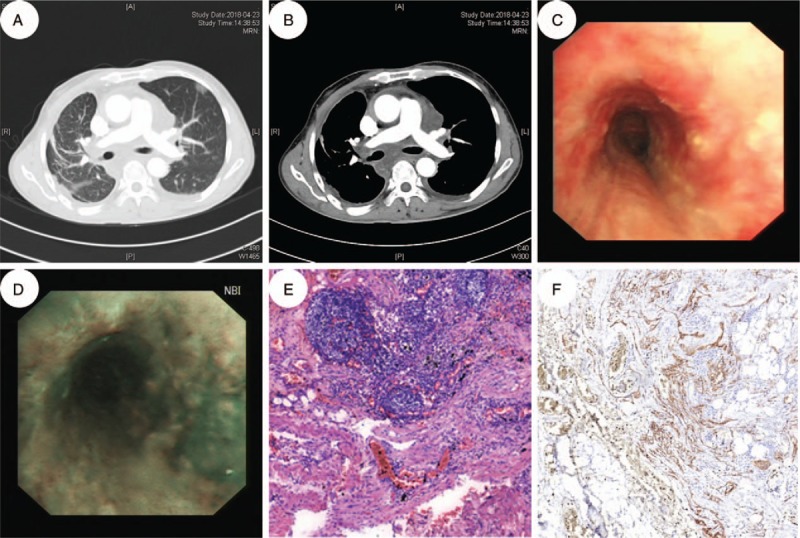
A–B, Chest CT demonstrates diffuse thickening of the interlobular septa, patch opacity and ground-glass lesions in both lungs (A); CT also reveals mediastinal enlargemengt without enhancement and bilateral pleural effusion (B). C–D, A bronchoscopy shows edematous changes and thin-walled translucent vesicles distributed in a diffuse way. E–F, Histology of the lung tissue showing proliferation of irregular lymphatic vessels, lined by single layer of endothelial cells lacking cytological atypia (E, HE, original magnification ×100; F, immunohistochemical staining with D2–40 revealing proliferative lymphatic channels, original magnification ×100).

A 59-year-old man with a 20-year history of liver cirrhosis and recurrent hepatic encephalopathy (HE), without significant etiology, presented with cough and sputum for 2 months. He had no history of trauma, surgery, or other meaningful history. Upon physical examination, he looked very thin but with no other positive signs. Laboratory examinations revealed severe microcytic hypochromic anemia, with hemoglobin value of 78 g/L and albumin value of 24 g/L. The value of blood ammonia was 171 μmol/L. The markers for tumor (CEA, CA-125, CA-199, and AFP), connective tissue disease and hepatitis were negative. Chest computed tomography (CT) revealed significant mediastinal enlargement in a liquid-like density without enhancement, peribronchovascular infiltrations, ground-glass lesions, and a small amount of effusion in the left pleural cavity (Fig. 1A and B). Bronchoscopy showed a smooth and edematous mucosal surface and diffuse thin walled translucent vesicles in the mucosa of both bronchi (Fig. 1C and D). We undertook transbronchial biopsy and bronchoaleolar lavage (BAL) in turn. However, symptoms of cough got worse suddenly accompanied by excessive chyloptysis after BAL. Emergency surgical intervention was done and surgical ligation of the thoracic duct was performed. Histologically, dilated, irregular, and anastomozing vascular structures positive for D2–40, which are markers of the vascular endothelium, were found (Fig. 1E and F). His symptoms improved significantly following surgery and low fat medium chain fat treatment and he was discharged uneventfully 10 days later. The patient has been followed up for 6 months and there is no relapse.

## 3 Discussion

DPL is a very rare benign lymphatic disorder^[1–3]^ and mainly reported in western countries. It usually presents in childhood and young adult patients.^[1]^ A PubMed search with the words “diffuse pulmonary lymphangiomatosis” found a total of 16 articles in English. Among the cases of DLP reported, the age of patients when DPL was diagnosed ranged from 1 month to 53 years old. To the best of our knowledge, the current case was the oldest one reported in the English literature.

The major causes of DPL included congenital factors^[4,5]^ and acquired diseases.^[6,7]^ Few studies reported the association between liver cirrhosis and DPL and hepatic lymphangiomatosis is not common.^[8]^ Our case was combined cirrhotic and hepatic encephalopathy for 20-year, with no obvious cause. It is puzzled to us that the symptoms of HE can be relieved without no treatment. Whether there is a correlation between liver cirrhosis and DPL needs further study.

The earlier symptom of DPL was atypical and imaging examination, even if non-specific, seems to be more important in diagnosing DPL. Chest radiograph of DPL typically shows enlarged mediastinum, diffuse peribronchovascular infiltrations, and bilateral pleural effusions.^[9,10]^ Our case showed suggestive feature of DPL, diffuse liquid-like infiltration in the mediastinal and hilar soft tissue, peribronchovascular and interlobular septal thickening, ground-glass opacities, and pleura effusion.^[9,10]^ Although CT features are highly suggestive of DPL in our case, the image diagnosis is still difficult especially for us who lack experience. It suggests that we need to raise awareness of the rare disease when characteristic imaging findings of DPL appear.

Definite diagnosis of DPL depends on pathological examination either by bronchoscopy, mediastinal biopsies, or open lung biopsy. Among the cases reported, few summarized the bronchoscopy manifestation of DPL. Among the 16 cases of histologically approved DLP reported in the literature, only 7 cases (43.75%) provided the data of bronchoscopy findings, including milky or chylous secretion (14.2%), hemorrhage (14.2%), thin walled translucent vesicle (28.5%),^[11,12]^ airway mucosal inflammatory changes (42.8%). Early bronchoscopy showing may be not specific and thin walled translucent vesicle containing milky fluid may be often seen in advanced cases,^[11]^ which may be suggestive for DPL.

Serious complications of bronchoscopy have not been reported in DPL. In our case a diagnostic pulmonary biopsy caused serious and life-threatening complications due to massive chyloptysis after bronchoscopy. We suspect that chyloptysis is due to the suddenly decreased pressure in lymphatic vessels after bronchoscopy. Chylothorax has been reported after open lung bioscopy^[13]^ or surgical removal of the mediastinal mass.^[14]^ We suggest that non-invasive methods for diagnosis should be first considered, especially in asymptomatic patients^[13]^ or in patients with severely enlarged mediastinum or with thin-walled translucent vesicle under endoscopy.

There are no specific treatments for DPL.^[15,16]^ Current therapies, including surgical interventions,^[17]^ systemic treatment (corticosteroids, immunomodulating agents, and chemotherapy^[18,19]^), radiation therapy,^[20]^ are essentially palliative to mainly alleviate clinical symptoms.^[2,3,16]^ The value of surgical resection is impaired by the difficulty to separate lymph collections from normal structures and high rates of recurrence.^[1,21]^ Thoracotomy or thoracoscopic surgery may be considered for localized intrapulmonary or mediastinal lesions, especially those with rapid growth.^[15,17,22]^ The symptoms of cough and sputum improved significantly in our patient after surgical intervention and medium-chain triglycerides diet therapy and no other treatment was used. Fortunately, following up for 6 months, no relapse was observed in our case.

The prognosis for patients with DPL is progressive with relatively indolent courses. Patients with more generalized disease appear to have a poorer outcome.^[1,3]^

In all, this case is unusual in many ways. Firstly, he was older than most patients described in the literature; secondly, to our knowledge, we are the first to report the serious complications of bronchoscopy in DPL; thirdly, short-term prognosis is good after surgical treatment in our case.

DPL is a rare disorder, with high lethality and poor prognosis, and definitive diagnosis requires lung biopsy. Chest x-ray/CT and bronchoscopy finding, even if non-specific, may be suggestive. Complications of bronchoscopy should be considered, if thin walled translucent vesicle under endoscopy was found. The current treatment is still based on symptomatic mitigation.

## Author contributions

**Data curation**: Wencheng Yu, Liyun Mi, Yunqing Chen.

**Formal analysis:** Haihong Gong.

**Investigation:** Wencheng Yu.

**Software:** Jinpeng Cong.

**Supervision:** Wencheng Yu, Haihong Gong.

**Writing – original draft:** Haihong Gong.

**Writing – review & editing:** Liyun Mi, Jinpeng Cong, Wei Cheng, Haihong Gong.
